# Free-Floating Iris Pigmented Epithelial Cyst in the Anterior Chamber

**DOI:** 10.1155/2016/4731037

**Published:** 2016-01-21

**Authors:** Tryfon Rotsos, Georgios Bagikos, Spyridon Christou, Chrysanthos Symeonidis, Thekla Papadaki, Ioannis Papaeuthimiou, Dimitrios Miltsakakis

**Affiliations:** ^1^1st Department of Ophthalmology, University of Athens, General Hospital “G. Gennimatas”, Mesogeion Avenue 158, 156 69 Athens, Greece; ^2^Department of Ophthalmology, General Hospital “G. Gennimatas”, Mesogeion Avenue 158, 156 69 Athens, Greece; ^3^2nd Department of Ophthalmology, Aristotle University of Thessaloniki, “Papageorgiou” General Hospital, Thessaloniki Ring Road, Macedonia, 564 03 Thessaloniki, Greece

## Abstract

An unusual case of a free-floating peripheral pigmented cyst in the anterior chamber is presented. A 30-year-old Caucasian male presented reporting a visual defect on his right eye in prone position over the past year. Slit-lamp examination revealed a small pigmented free-floating peripheral iris cyst at the 6 o'clock position in the anterior chamber. Ultrasound biomicroscopy revealed an unfixed epithelial pigmented cyst with an extremely thin wall and no internal reflectivity. Due to the lack of severity of visual disturbance of the patient, no surgical treatment was indicated. The patient is to be followed up annually and advised to return immediately in case of pain or any visual symptoms. Free-floating iris cysts in the anterior chamber are uncommon and remain stable in the majority of cases. Management includes only regular observation until any complications arise.

## 1. Introduction

A cyst is defined as an epithelial-lined cavity. A primary iris pigment epithelium cyst is an epithelial-lined cavity which involves a portion of the iris and which has no recognizable etiology [1]. A secondary iris cyst is an epithelial-lined space which involves a portion of the iris and which has a recognizable etiology, such as surgical trauma, nonsurgical trauma, or miotic drugs [1, 2].

A review of the available literature indicated that cysts may occur on the pupillary margin, in the posterior epithelial layer of the iris, or as free-floating cysts in the anterior chamber or in the vitreous cavity [2]. Finally, in rare cases, a cyst may be associated with the iris stroma [2].

Pigmented cysts in the anterior chamber have been previously described in the relevant literature. Coats in 1912 reported a case in which the cyst shifted with movement of the head [1]. Yanoff and Zimmerman in 1965 described a case in which the involved eye was enucleated because of the suspected melanoma and reviewed the literature on the subject [1]. Donaldson in 1967 presented clinical photographs of two cases [1]. Fine reported a free-floating cyst in the anterior chamber which resulted in decreased visual acuity and required surgical removal [1]. These anterior chamber cysts appear to be lined by iris pigment epithelium and to contain clear fluid. In one case, the cyst was lined by both pigmented and nonpigmented epithelium [1].

This case report describes a case of a free-floating iris cyst in the anterior chamber. Potential complications and management of free-floating iris cysts are also discussed.

## 2. Case Report

**Figure 1:**
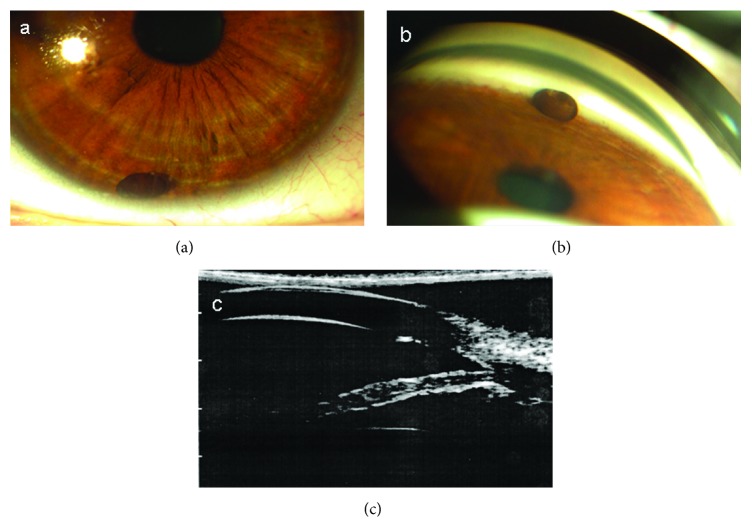
(a) Free-floating peripheral iris cyst at 6 o'clock (primary gaze). (b) Gonioscopy revealing an open angle. (c) Ultrasound biomicroscopy of the right eye.

A 30-year-old Caucasian male was referred reporting a visual defect on his right eye in prone position over the previous year. Slit-lamp examination revealed a small pigmented free-floating peripheral iris cyst of approximately 1.2 by 2 mm at the 6 o'clock position in the anterior chamber (Figure 1(a)). The cyst remained unchanged during the previous year. Mobilization of the cyst occurred with head tilt but never caused pain or visual acuity decrease. There was no personal history of ocular diseases, surgery, and trauma or systemic diseases. Moreover, there was no family history of ocular or systemic diseases and allergies. Best-corrected visual acuity was 20/20 in both eyes while intraocular pressure was 13 mmHg and 15 mmHg. Refractive media were translucent. Gonioscopy showed an open anterior chamber angle in all quadrants (Figure 1(b)). Fundoscopy revealed no pathological findings. Blood tests were within normal limits. Ultrasound biomicroscopy (UBM) revealed an unfixed epithelial pigmented cyst with an extremely thin wall and no internal reflectivity (Figure 1(c)). The device used was a P60 UBM with a 35-MHz probe (Paradigm Medical Industries, Salt Lake City, UT, USA).

## 3. Discussion

Primary iris cysts are divided into epithelial and stromal, with each having different clinical characteristics. Primary iris pigment epithelial cysts arise between the pigmented epithelial layers of the iris and occur at the pupillary margin (central cysts), in the midportion of the iris (midzonal cysts), or, more commonly, in the iridociliary sulcus (peripheral cysts) [3–5]. In some cases, the cysts are released free from their epithelial attachment and migrate into the anterior chamber or vitreous chamber (dislodged cysts) [3, 6, 7]. Primary stromal cysts occur within the iris stroma and are not directly continuous with the posterior epithelium. They apparently arise from ectopic surface epithelium which is entrapped in the iris during embryologic development [3, 6, 7].

A study of the natural course and complications of these lesions has shown that the great majority of primary iris cysts, particularly those which arise from the iris pigment epithelial layer, are stationary lesions which rarely progress or cause visual complications. The natural course of primary epithelial cysts differs from that of secondary iris cysts which are secondary to surgical or nonsurgical trauma. The latter lesions commonly increase in size and result in complications such as angle closure glaucoma, plateau iris syndrome, and secondary pigment dispersion syndrome [3, 4, 6]. However, these complications are uncommon.

The major clinical importance of primary iris cysts lies in their similarity to neoplasms of the iris and ciliary body [3, 6, 7]. It is concluded that the majority of primary iris cysts require no treatment, unless they are associated with ocular complications.

Free-floating iris cysts comprise <1% of primary iris pigment epithelial cysts. The differential diagnosis includes iris stromal cyst, iris or ciliary body melanoma, adenoma of the iris pigment epithelium, and medulloepithelioma [3, 4, 7]. UBM can be used to distinguish iris cysts from these uveal tumours.

Management of epithelial cysts includes observation until complications are observed. Numerous approaches have been used to excise epithelial cysts, but currently a wide excision of the intact cyst is preferred. If the cyst is adherent to any intraocular structures, it may be aspirated prior to excision. Photocoagulation of epithelial cysts, a less invasive procedure than surgical removal, has been performed successfully. Photocoagulation is less effective when the cyst is nonpigmented or adherent to underlying structures [3, 5, 6].

Management options for epithelial proliferation include

- cryopexy of the involved corneal surface to close the wound gape or fistula;
- resecting the posterior membrane;
- intracameral injection of 5-fluorouracil [3, 5, 6].

Management of glaucoma is a challenge and has a high failure rate using traditional filtration surgery techniques. Glaucoma drainage tube implants have been shown to be the most effective procedure, with both fibrous and epithelial ingrowth [3]. Cycloablation is used only when other treatment modalities fail [3].

Due to the lack of severity of visual disturbance of the patient, no surgical treatment was indicated. The patient is to be followed up annually and advised to return immediately in case of pain or any visual symptoms. Free-floating iris cysts in the anterior chamber are uncommon and remain stable in their majority. Management includes only regular observation until any complications arise.
